# Cardioembolic Stroke in Young: A Case of Atrial Myxoma Origin

**DOI:** 10.7759/cureus.27890

**Published:** 2022-08-11

**Authors:** Hiba Salam, Mohith K Reddy, V H Ganaraja, Sashikala V, Suresha Kodapala

**Affiliations:** 1 Neurology, Vydehi Institute of Medical Sciences and Research Centre, Bangalore, IND; 2 General Medicine, Vydehi Institute of Medical Sciences and Research Centre, Bangalore, IND; 3 Pathology, Vydehi Institute of Medical Sciences and Research Centre, Bangalore, IND

**Keywords:** stroke in young, cardioembolic stroke, echocardiography, atrial myxoma, posterior circulation stroke

## Abstract

Stroke is one of the leading causes of mortality and disability. It can be rarely caused by cardiac myxoma. Sometimes stroke may be its first clinical manifestation. Here we report a case of posterior circulation stroke in left atrial myxoma. A 45-year-old female patient presented with a history of recurrent episodes of dizziness and headache of three months duration. Neurological examination showed impaired tandem gait. Magnetic resonance imaging (MRI) of the brain revealed infarction in the left posteroinferior cerebellar hemisphere. Echocardiography of the patient revealed a large left atrial mass suggestive of atrial myxoma and an ejection fraction of 60%.

The patient was operated on for atrial myxoma two days after the diagnosis, and histopathology confirmed the diagnosis. Postoperatively she remained well and was managed on anti-platelet drugs. Atrial myxoma should be considered as a possible differential while evaluating a case of cardioembolic stroke, and echocardiography detects the presence of an atrial myxoma. It is also essential that atrial myxomas are managed early to prevent recurrent strokes.

## Introduction

Stroke is one of the leading causes of mortality and a major cause of disability [1]. It occurs when there is a disruption in the blood supply to the brain, causing damage to a part of the brain [1]. Cardioembolic stroke is a type of ischemic stroke, accounting for 20% of total stroke cases [2]. Though atrial fibrillation is attributed to be one of the most common causes of cardioembolic stroke, various other causes include acute myocardial infarction, infective endocarditis, valvular heart disease, and cardiac myxoma [3]. Cardiac myxoma, though a rare cause of cardioembolic stroke, is the most common primary cardiac tumour, accounting to 0.5% of cardiac emboli [3]. It is also considered to be an important etiology of stroke in young [3]. Identifying the etiology, and surgical removal along with medical management is essential for treating and preventing the recurrence of thromboembolic events [2]. The neurological manifestations of stroke include sudden weakness, numbness and signs of paralysis, cranial nerve deficits, difficulty in speech, visual disturbances, dizziness, and headache [4]. Hereby, we describe a case of posterior circulation stroke in a patient with left atrial myxoma.

## Case presentation

A 45-year-old female patient presented with recurrent episodes of giddiness for three months. Following an episode of dizziness three months back, associated with transient loss of vision, she developed weakness in the left upper and lower limb. This was followed by complete spontaneous recovery of her symptoms within a week of onset. This episode was not associated with loss of consciousness, sensory impairment, or cranial nerve deficits. Sometimes dizziness episodes were also associated with headache, which was localized to the occipital region, not associated with vomiting. There was a history of chest pain and palpitations for six months. The pain was squeezing type, mainly in the epigastric region, and used to worsen with walking for 15 to 20 minutes and relieved with rest. There were no other significant medical history or vascular risk factors.

On examination, the patient was alert and responsive to oral commands. Her vital parameters were within normal limits, with a heart rate of 86/minute and regular. Blood pressure was 122/86mmHg in the right upper limb in the supine position. Neurological examination showed normal higher mental functions with no motor, sensory, cranial nerve deficits, meningeal signs except mild impairment in tandem gait while walking. There was no nystagmus. Finger nose test was normal bilaterally. Cardiovascular and other systemic examination was found to be normal.

**Figure 1:**
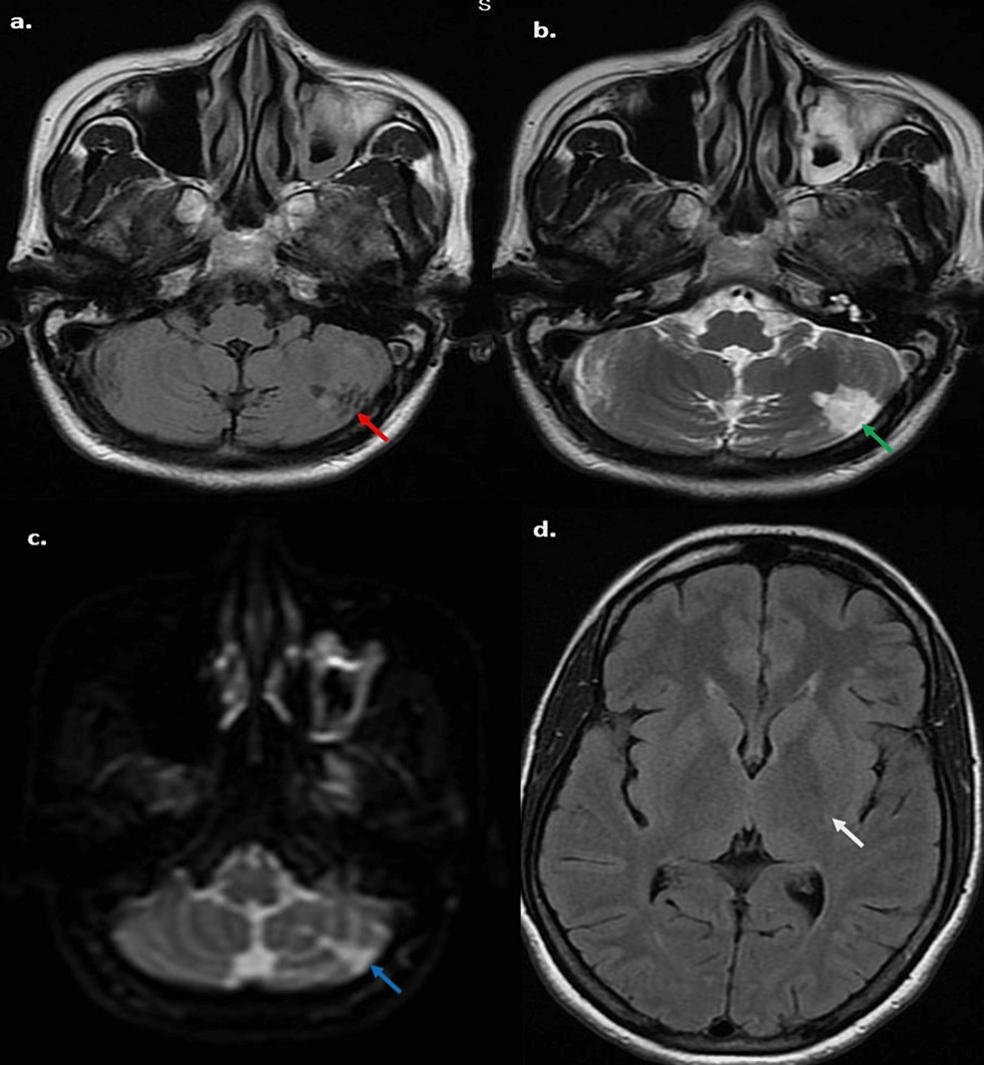
MRI of the brain (a) FLAIR axial sequence showing hypointensity in the left cerebellar hemisphere(red arrow), (b) T2 axial sequence showing hyperintensity in the left cerebellar hemisphere(green arrow), (c) MRI diffusion sequence showing diffusion restriction in the same region (blue arrow) with overall features suggesting infarction in the left cerebellar hemisphere. (d) MRI brain showing normal supratentorial structures (white arrow). MRI: magnetic resonance imaging, FLAIR: fluid-attenuated inversion recovery.

Her routine laboratory investigations - complete hemogram, liver and kidney function tests, blood glucose, coagulation profile, and serum electrolytes were within normal ranges. Erythrocyte sedimentation rate (ESR) was 15 mm/hour. Serological markers for HIV, Hepatitis B, and Hepatitis C were negative. Magnetic resonance imaging (MRI) of the brain showed features suggesting infarction in the left posteroinferior cerebellar hemisphere (Figure 1).

**Figure 2:**
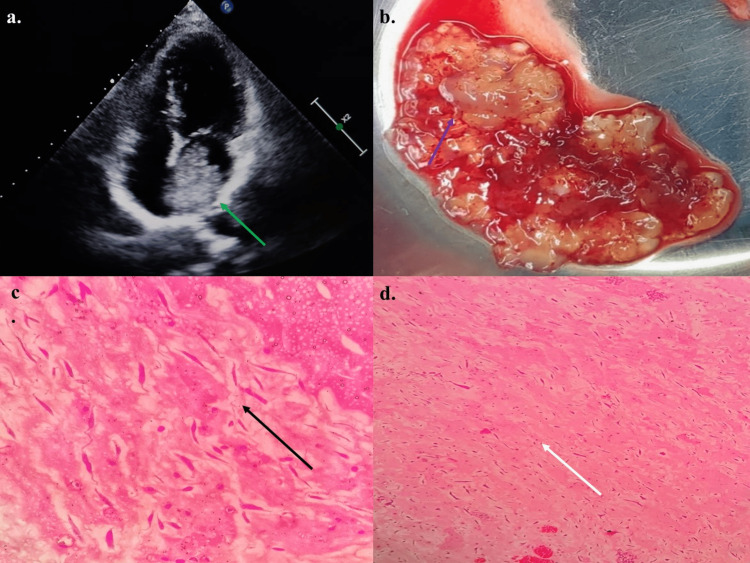
(a) Echocardiography showing a large left atrial mass (green arrow), (b) intraoperative observation of a dirty white gelatinous mass which was attached to the interatrial septum in the left atrium (purple arrow), (c) light microscopy of the excised tissue showing loose myxoid stroma with scattered spindle cells (black arrow), and (d) scattered round, polygonal or stellate cells with dense irregular nuclei and spindle cells in myxoid stroma(white arrow)with overall features suggesting atrial myxoma.

Her echocardiography revealed a 3.2 * 3.6 cm large left atrial mass suggestive of atrial myxoma and left ventricular ejection fraction of 60%. She underwent surgical resection for atrial myxoma. Intraoperatively, a gelatinous mass was attached to the interatrial septum in the left atrium, and histopathological findings were consistent with atrial myxoma (Figure 2).

Postoperatively she remained well. Later she was managed with anti-platelet agents for stroke.

## Discussion

Hereby, we report an unusual presentation of atrial myxoma, presenting as a case of posterior circulation stroke in young. Though cardioembolic stroke in young is common, however myxoma as a cause of stroke is uncommon. This patient presented with recurrent episodes of dizziness, headache, chest pain and palpitations, and was treated by surgical removal of the myxoma along with medical management.

Atrial myxoma, the most common primary cardiac tumor, accounting for 0.5% of cardiac emboli, arises from the multipotent mesenchymal cell of the endometrium [5]. The etiology of atrial myxoma is currently unknown, and most cases are sporadic and benign. It usually presents as a diagnostic triad. The patients may present with obstructive symptoms, constitutional symptoms, or embolic phenomenon [6,7]. Obstructive symptoms include symptoms of heart failure, dyspnoea, syncope, and rarely sudden death. Constitutional symptoms may also present with myalgia, arthralgia, weight loss, fatigue, fever, Raynaud’s phenomenon, and finger clubbing which may sometime mimic autoimmune disease or vasculitis [7]. Though elevation of erythrocyte sedimentation rate, C-reactive protein with anemia, and hyperglobulinemia is often seen in active illness, magnetic resonance imaging (MRI) of the brain and trans-oesophageal echocardiography are the diagnostic imaging modality of choice [2,8]. These myxomatous emboli can rarely cause aneurysms by infiltrating the vessel walls [9]. Annual follow-up is recommended with echocardiography for three to four years of post-surgical removal due to the chances of recurrence and also to look for metastasis [10]. Standard management protocol for treating atrial myxoma includes prompt surgical resection with the examination of the cardiac chambers and valves. However, chemoradiotherapy is a few suggested treatment modalities for its metastasis [2,10].

## Conclusions

Cardioembolic stroke in young secondary to atrial myxoma is rare. This case demonstrates the importance of considering atrial myxoma as a possible differential while evaluating a case of cardioembolic stroke and reveals the importance of doing relevant investigations in a stroke patient for ruling out cardiac causes of stroke. It also demonstrates the importance of early management of atrial myxomas to prevent recurrent strokes.

Early surgical management of atrial myxoma and long-term follow-up after surgery is often required in those who present with stroke to look for metastasis. We present this case to emphasize the importance of early diagnosis and basic cardiac evaluation of secondary causes of stroke and also the need for long-term close follow-up.
